# Crystal structure of bis­(1-methyl-1*H*-imidazole-κ*N* ^3^)(5,10,15,20-tetra­phenyl­porphyrinato-κ^4^ *N*)iron(II)–1-methyl-1*H*-imidazole (1/2)

**DOI:** 10.1107/S2056989015002364

**Published:** 2015-02-11

**Authors:** Ye Guan, Douglas R. Powell, George B. Richter-Addo

**Affiliations:** a Department of Chemistry and Biochemistry, University of Oklahoma, 101 Stephenson Pkwy, Norman, OK 73019, USA

**Keywords:** crystal structure, model porphyrins, Fe^II^ complex

## Abstract

The title compound, [Fe(C_44_H_28_N_4_)(C_4_H_6_N_2_)_2_]·2C_4_H_6_N_2_, is a six-coordinate Fe^II^–porphyrinate complex with the metal located on a center of inversion and coordinated by two axial 1-methyl­imidazole ligands; the complex crystallizes as a 1-methyl­imidazole disolvate. The 1-methyl­imidazole group bonded to the Fe^II^ atom [occupancy ratio 0.789 (4):0.211 (4)] and the unbound 1-methyl­imidazole mol­ecule [0.519 (4):0.481 (4)] were disordered. The average Fe—N(porphyrinate) bond length is 1.998 (3) Å and the axial Fe—N(imidazole) bond length is 1.9970 (12) Å. In the crystal, mol­ecules are linked into a three-mol­ecule aggregate by two weak C—H⋯N inter­actions.

## Related literature   

For the function and structure of bis-histidine-coordinated cytochromes b, see: Xia *et al.* (1997 ▸). For the structures of other models of bis-histidine-coordinated hemes in proteins, see: Walker (2004 ▸). For the parallel and perpendicular orientation preferences of imidazole ligands in model porphyrins, see: Safo *et al.* (1991 ▸). For the synthesis of some bis-imidazole complexes, see: Higgins *et al.* (1991 ▸). The structure of Fe(TPP)(1-MeIm)_2_ (*i.e.* the solvate-free title compound) was briefly mentioned in a meeting abstract, but no structural information is available, see: Steffen *et al.* (1978 ▸). For an example of a complex with a low-spin ferrous center, see: Scheidt & Reed (1981 ▸).
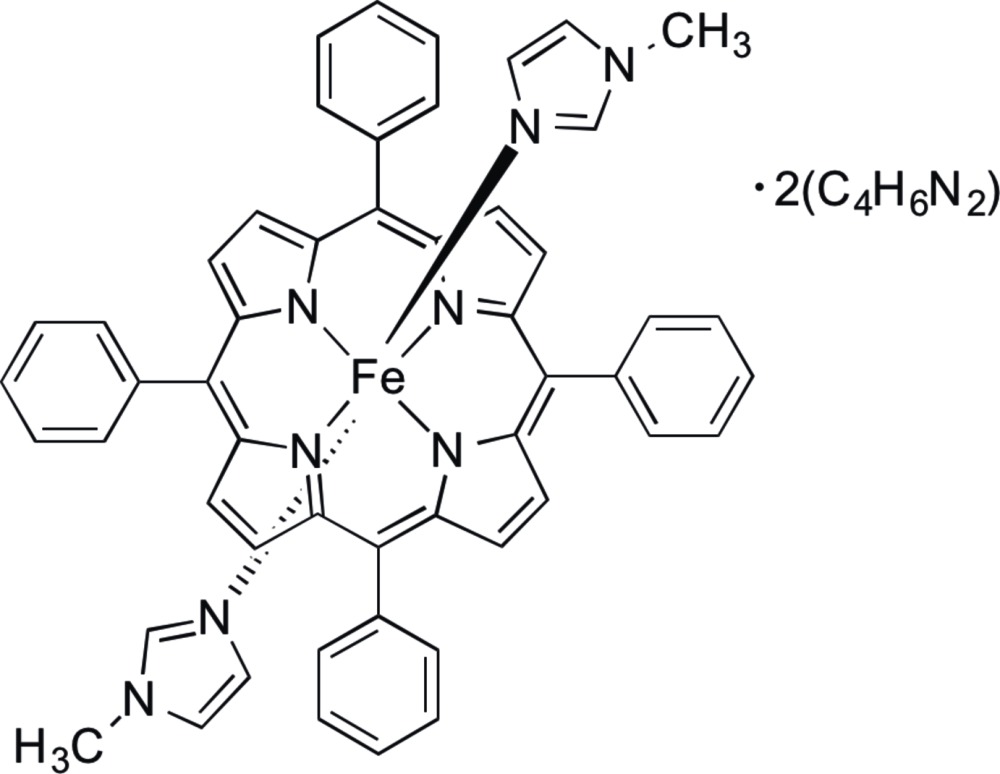


## Experimental   

### Crystal data  

[Fe(C_44_H_28_N_4_)(C_4_H_6_N_2_)_2_]·2C_4_H_6_N_2_
*M* *_r_* = 996.98
Triclinic, 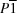

*a* = 9.3108 (4) Å
*b* = 10.1943 (4) Å
*c* = 13.4745 (5) Å
α = 81.557 (2)°
β = 79.143 (2)°
γ = 77.797 (3)°
*V* = 1220.03 (9) Å^3^
*Z* = 1
Mo *K*α radiation
μ = 0.37 mm^−1^
*T* = 100 K
0.26 × 0.26 × 0.04 mm

### Data collection  

Bruker APEX CCD diffractometer
Absorption correction: multi-scan (*SADABS*; Bruker, 2002 ▸) *T*_min_ = 0.911, *T*_max_ = 0.986
18378 measured reflections
6043 independent reflections
5263 reflections with *I* > 2σ(*I*)
*R*_int_ = 0.018

### Refinement  

*R*[*F* ^2^ > 2σ(*F* ^2^)] = 0.040
*wR*(*F* ^2^) = 0.119
*S* = 1.00
6043 reflections
432 parameters
377 restraints
H-atom parameters constrained
Δρ_max_ = 0.43 e Å^−3^
Δρ_min_ = −0.36 e Å^−3^

### 

Data collection: *SMART* (Bruker, 2007 ▸); cell refinement: *SAINT* (Bruker, 2007 ▸); data reduction: *SAINT*; program(s) used to solve structure: *SHELXTL* (Sheldrick, 2015 ▸); program(s) used to refine structure: *SHELXTL*; molecular graphics: *SHELXTL*; software used to prepare material for publication: *SHELXTL*.

## Supplementary Material

Crystal structure: contains datablock(s) I, New_Global_Publ_Block. DOI: 10.1107/S2056989015002364/tk5355sup1.cif

Structure factors: contains datablock(s) I. DOI: 10.1107/S2056989015002364/tk5355Isup2.hkl

Supporting information file. DOI: 10.1107/S2056989015002364/tk5355Isup3.cdx

Supporting information file. DOI: 10.1107/S2056989015002364/tk5355Isup4.docx

**Fig. 1.**
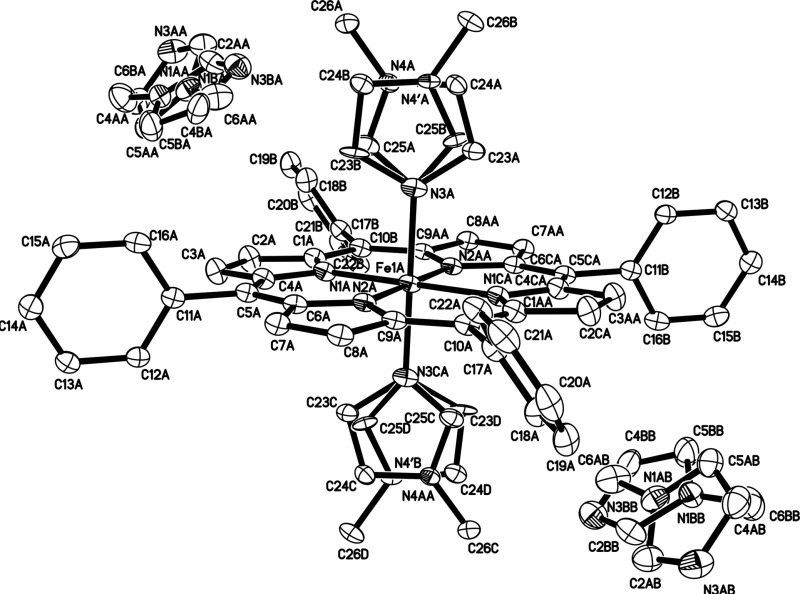
The molecular structure of the title compound showing atom-labeling scheme and displacement ellipsoids drawn at the 50% probability level. H atoms have been omitted for clarity.

. DOI: 10.1107/S2056989015002364/tk5355fig1.tif
The mol­ecular structure of the title compound showing atom-labeling scheme and displacement ellipsoids drawn at the 50% probability level. H atoms have been omitted for clarity.

CCDC reference: 1047218

Additional supporting information:  crystallographic information; 3D view; checkCIF report

## Figures and Tables

**Table 1:** Hydrogen-bond geometry (, )

*D*H*A*	*D*H	H*A*	*D* *A*	*D*H*A*
C19H19N3*A* ^i^	0.95	2.55	3.478(4)	164

Symmetry code: (i) 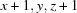.

## supplementary crystallographic information

### S1. Introduction

Bis-histidine coordinated hemes are present in a number of cytochrome *b* complexes, and are known to be involved in electron transfer processes (Xia *et al.*, 1997). The parallel and perpendicular relative orientations of the histidine ligands are believed to have correlations with the spectroscopic properties of the proteins (Walker, 2004). As models of bis-histidine coordinated cytochrome *b*, several cationic bis-imidazole coordinated porphyrin complexes have been synthesized and their structures have been determined (Safo *et al.*, 1991). Here, we report the molecular structure of a neutral bis-imidazole coordinated Fe^II^ complex, Fe(TPP)(1-MeIm)_2_^.^2(1-MeIm). The molecular structure of the titled compound is shown in Fig. 1. The porphyrin complex was located on an inversion center. The 1-methyl­imidazole groups bonded to the metal and the unbound 1-methyl­imidazoles were disordered. The average Fe—Np bond distance is 1.998 (3) Å and the axial Fe—N_Im_ distance is 1.9970 (12) Å, suggesting a low-spin ferrous center (Scheidt & Reed, 1981). The two 1-MeIm planes are mutually parallel. The projection of the axial ligand has a dihedral angle of 28.04 (15)° with the closest Fe—Np bond.

### S2. Experimental

The Fe(TPP)(1-MeIm)_2_^.^2(1-MeIm) was obtained serendipitously as follows: To a 10 ml CH_2_Cl_2_ solution of (TPP)FeCl (0.010 g, 0.014 mmol) was added N-hy­droxy­amphetamine (7 mg, 46.3 mmol) and 1-MeIm (0.05 ml, 29.0 mmol) under nitro­gen. The color of the solution changed from brown to reddish purple during a 6 h period. The solution was dried under reduced pressure. The residue was dissolved in CH_2_Cl_2_ and filtered, and an equal volume of hexane was added. A red plate shaped crystal grew from the slow evaporation of this mixture at room temperature under nitro­gen.

### S3. Refinement

H atoms were located geometrically and treated as riding on their parent atoms with C—H = 0.95 Å for aromatic and 0.98 Å for aliphatic, with *U*_iso_(H) = 1.2–1.5*U*_eq_(C). The 1-methyl­imidazole groups bonded to the metal were disordered. The occupancies of the metal bound ligands were refined to 0.789 (4) and 0.211 (4) for the unprimed and primed atoms. The occupancies of the unbound imidazoles refined to 0.519 (4) and 0.481 (4) for the A and B molecules.

### Crystal data

**Table:**

[Fe(C_44_H_28_N_4_)(C_4_H_6_N_2_)_2_]·2C_4_H_6_N_2_	*Z* = 1
*M**_r_* = 996.98	*F*(000) = 522
Triclinic, *P*1	*D*_x_ = 1.357 Mg m^−^^3^
*a* = 9.3108 (4) Å	Mo *K*α radiation, λ = 0.71073 Å
*b* = 10.1943 (4) Å	Cell parameters from 8102 reflections
*c* = 13.4745 (5) Å	θ = 2.3–28.3°
α = 81.557 (2)°	µ = 0.37 mm^−^^1^
β = 79.143 (2)°	*T* = 100 K
γ = 77.797 (3)°	Plate, red
*V* = 1220.03 (9) Å^3^	0.26 × 0.26 × 0.04 mm

### Data collection

**Table:**

Bruker APEX CCD diffractometer	5263 reflections with *I* > 2σ(*I*)
φ and ω scans	*R*_int_ = 0.018
Absorption correction: multi-scan (*SADABS*; Bruker, 2002)	θ_max_ = 28.3°, θ_min_ = 1.6°
*T*_min_ = 0.911, *T*_max_ = 0.986	*h* = −12→12
18378 measured reflections	*k* = −13→13
6043 independent reflections	*l* = −17→17

### Refinement

**Table:**

Refinement on *F*^2^	Primary atom site location: structure-invariant direct methods
Least-squares matrix: full	Secondary atom site location: difference Fourier map
*R*[*F*^2^ > 2σ(*F*^2^)] = 0.040	Hydrogen site location: mixed
*wR*(*F*^2^) = 0.119	H-atom parameters constrained
*S* = 1.00	*w* = 1/[σ^2^(*F*_o_^2^) + (0.071*P*)^2^ + 0.5*P*] where *P* = (*F*_o_^2^ + 2*F*_c_^2^)/3
6043 reflections	(Δ/σ)_max_ < 0.001
432 parameters	Δρ_max_ = 0.43 e Å^−^^3^
377 restraints	Δρ_min_ = −0.36 e Å^−^^3^

### Special details

**Table:**

Geometry. All e.s.d.'s (except the e.s.d. in the dihedral angle between two l.s. planes) are estimated using the full covariance matrix. The cell e.s.d.'s are taken into account individually in the estimation of e.s.d.'s in distances, angles and torsion angles; correlations between e.s.d.'s in cell parameters are only used when they are defined by crystal symmetry. An approximate (isotropic) treatment of cell e.s.d.'s is used for estimating e.s.d.'s involving l.s. planes.

### Fractional atomic coordinates and isotropic or equivalent isotropic displacement parameters (Å^2^)

**Table:**

	*x*	*y*	*z*	*U*_iso_*/*U*_eq_	Occ. (<1)
Fe1	0.5000	0.5000	0.5000	0.02574 (10)	
N1	0.52081 (14)	0.45129 (13)	0.35915 (9)	0.0280 (3)	
N2	0.60381 (13)	0.31382 (13)	0.54440 (9)	0.0261 (2)	
N3	0.30392 (14)	0.44052 (13)	0.53811 (10)	0.0310 (3)	
N4	0.1140 (2)	0.3434 (2)	0.54313 (16)	0.0257 (5)	0.789 (4)
N4'	0.0828 (6)	0.3902 (8)	0.5888 (6)	0.0247 (14)	0.211 (4)
C1	0.47503 (17)	0.53338 (16)	0.27524 (11)	0.0313 (3)	
C2	0.5222 (2)	0.46249 (18)	0.18604 (12)	0.0395 (4)	
H2	0.5055	0.4974	0.1190	0.047*	
C3	0.5942 (2)	0.33746 (18)	0.21543 (12)	0.0385 (4)	
H3	0.6378	0.2678	0.1732	0.046*	
C4	0.59236 (17)	0.33016 (16)	0.32344 (11)	0.0304 (3)	
C5	0.65568 (16)	0.21560 (16)	0.38157 (11)	0.0285 (3)	
C6	0.66021 (16)	0.21019 (15)	0.48511 (11)	0.0276 (3)	
C7	0.73309 (17)	0.09311 (16)	0.54325 (12)	0.0317 (3)	
H7	0.7799	0.0087	0.5195	0.038*	
C8	0.72186 (17)	0.12684 (16)	0.63825 (12)	0.0305 (3)	
H8	0.7607	0.0710	0.6937	0.037*	
C9	0.63976 (15)	0.26358 (15)	0.63937 (11)	0.0265 (3)	
C10	0.60348 (16)	0.33397 (16)	0.72504 (11)	0.0291 (3)	
C11	0.72561 (17)	0.09204 (15)	0.32906 (11)	0.0288 (3)	
C12	0.87028 (17)	0.07895 (16)	0.27499 (11)	0.0307 (3)	
H12	0.9256	0.1479	0.2729	0.037*	
C13	0.93466 (18)	−0.03372 (17)	0.22411 (12)	0.0329 (3)	
H13	1.0330	−0.0411	0.1871	0.040*	
C14	0.85489 (19)	−0.13543 (17)	0.22755 (13)	0.0360 (3)	
H14	0.8979	−0.2120	0.1921	0.043*	
C15	0.7121 (2)	−0.12480 (17)	0.28294 (15)	0.0398 (4)	
H15	0.6581	−0.1952	0.2867	0.048*	
C16	0.64794 (18)	−0.01155 (17)	0.33296 (13)	0.0358 (3)	
H16	0.5498	−0.0048	0.3703	0.043*	
C17	0.65558 (17)	0.26479 (16)	0.82149 (11)	0.0322 (3)	
C18	0.7494 (2)	0.32039 (19)	0.86635 (13)	0.0405 (4)	
H18	0.7780	0.4031	0.8359	0.049*	
C19	0.8014 (2)	0.2564 (2)	0.95494 (13)	0.0466 (4)	
H19	0.8639	0.2960	0.9850	0.056*	
C20	0.7619 (2)	0.1348 (2)	0.99913 (13)	0.0466 (5)	
H20	0.7983	0.0904	1.0591	0.056*	
C21	0.6695 (2)	0.0782 (2)	0.95580 (13)	0.0440 (4)	
H21	0.6427	−0.0053	0.9861	0.053*	
C22	0.61509 (18)	0.14289 (18)	0.86782 (12)	0.0370 (4)	
H22	0.5503	0.1039	0.8393	0.044*	
C23	0.1866 (3)	0.4950 (3)	0.6130 (3)	0.0304 (6)	0.789 (4)
H23	0.1886	0.5622	0.6544	0.037*	0.789 (4)
C24	0.0689 (3)	0.4316 (3)	0.6144 (2)	0.0301 (5)	0.789 (4)
H24	−0.0254	0.4467	0.6569	0.036*	0.789 (4)
C25	0.2530 (3)	0.3543 (3)	0.4999 (3)	0.0332 (7)	0.789 (4)
H25	0.3085	0.3039	0.4466	0.040*	0.789 (4)
C26	0.0300 (2)	0.2484 (2)	0.52161 (16)	0.0318 (5)	0.789 (4)
H26A	0.0482	0.1645	0.5671	0.048*	0.789 (4)
H26B	0.0619	0.2291	0.4508	0.048*	0.789 (4)
H26C	−0.0765	0.2878	0.5325	0.048*	0.789 (4)
C23'	0.2829 (11)	0.3246 (9)	0.4974 (10)	0.0282 (18)	0.211 (4)
H23'	0.3601	0.2725	0.4546	0.034*	0.211 (4)
C24'	0.1416 (9)	0.2948 (10)	0.5251 (7)	0.0319 (15)	0.211 (4)
H24'	0.0990	0.2277	0.5054	0.038*	0.211 (4)
C25'	0.1766 (7)	0.4706 (11)	0.5934 (8)	0.0269 (16)	0.211 (4)
H25'	0.1512	0.5434	0.6340	0.032*	0.211 (4)
C26'	−0.0660 (7)	0.4117 (8)	0.6480 (6)	0.0344 (19)	0.211 (4)
H26D	−0.1391	0.4115	0.6044	0.052*	0.211 (4)
H26E	−0.0864	0.4989	0.6754	0.052*	0.211 (4)
H26F	−0.0729	0.3393	0.7042	0.052*	0.211 (4)
N1A	0.1929 (6)	0.3111 (4)	0.1418 (4)	0.0464 (10)	0.519 (4)
C2A	0.0604 (5)	0.3741 (5)	0.1145 (4)	0.0522 (11)	0.519 (4)
H2A	−0.0295	0.3924	0.1613	0.063*	0.519 (4)
N3A	0.0707 (4)	0.4066 (4)	0.0165 (3)	0.0571 (10)	0.519 (4)
C4A	0.2185 (14)	0.3640 (11)	−0.0224 (9)	0.057 (2)	0.519 (4)
H4A	0.2606	0.3741	−0.0924	0.069*	0.519 (4)
C5A	0.2950 (7)	0.3056 (4)	0.0542 (4)	0.0515 (11)	0.519 (4)
H5A	0.3984	0.2684	0.0482	0.062*	0.519 (4)
C6A	0.2199 (9)	0.2633 (8)	0.2447 (6)	0.062 (2)	0.519 (4)
H6AA	0.1252	0.2752	0.2917	0.094*	0.519 (4)
H6AB	0.2865	0.3149	0.2634	0.094*	0.519 (4)
H6AC	0.2662	0.1673	0.2486	0.094*	0.519 (4)
N1B	0.2220 (6)	0.2984 (4)	0.0878 (4)	0.0465 (10)	0.481 (4)
C2B	0.1079 (7)	0.3523 (6)	0.1580 (4)	0.0498 (12)	0.481 (4)
H2BB	0.0230	0.4153	0.1408	0.060*	0.481 (4)
N3B	0.1271 (5)	0.3090 (4)	0.2505 (3)	0.0518 (10)	0.481 (4)
C4B	0.2654 (9)	0.2239 (8)	0.2407 (7)	0.0585 (19)	0.481 (4)
H4B	0.3130	0.1774	0.2958	0.070*	0.481 (4)
C5B	0.3215 (5)	0.2170 (6)	0.1430 (4)	0.0681 (16)	0.481 (4)
H5B	0.4143	0.1648	0.1164	0.082*	0.481 (4)
C6B	0.2291 (16)	0.3218 (12)	−0.0212 (9)	0.069 (3)	0.481 (4)
H6BA	0.1719	0.4116	−0.0396	0.104*	0.481 (4)
H6BB	0.1872	0.2531	−0.0440	0.104*	0.481 (4)
H6BC	0.3332	0.3166	−0.0540	0.104*	0.481 (4)

### Atomic displacement parameters (Å^2^)

**Table:**

	*U*^11^	*U*^22^	*U*^33^	*U*^12^	*U*^13^	*U*^23^
Fe1	0.02133 (15)	0.03238 (17)	0.02061 (15)	−0.00538 (11)	−0.00410 (10)	0.00776 (11)
N1	0.0253 (6)	0.0330 (6)	0.0222 (6)	−0.0041 (5)	−0.0041 (5)	0.0066 (5)
N2	0.0219 (5)	0.0335 (6)	0.0206 (5)	−0.0063 (5)	−0.0041 (4)	0.0066 (5)
N3	0.0250 (6)	0.0384 (7)	0.0268 (6)	−0.0077 (5)	−0.0072 (5)	0.0118 (5)
N4	0.0218 (9)	0.0272 (11)	0.0262 (10)	−0.0026 (7)	−0.0042 (7)	0.0007 (8)
N4'	0.017 (2)	0.030 (3)	0.028 (3)	−0.010 (2)	−0.005 (2)	0.003 (2)
C1	0.0313 (7)	0.0370 (8)	0.0219 (7)	−0.0047 (6)	−0.0048 (6)	0.0066 (6)
C2	0.0497 (10)	0.0407 (9)	0.0213 (7)	0.0009 (7)	−0.0058 (7)	0.0051 (6)
C3	0.0465 (9)	0.0403 (9)	0.0228 (7)	0.0003 (7)	−0.0055 (7)	0.0024 (6)
C4	0.0299 (7)	0.0351 (8)	0.0231 (7)	−0.0045 (6)	−0.0046 (5)	0.0047 (6)
C5	0.0245 (7)	0.0334 (7)	0.0257 (7)	−0.0057 (5)	−0.0050 (5)	0.0042 (6)
C6	0.0234 (6)	0.0316 (7)	0.0260 (7)	−0.0058 (5)	−0.0068 (5)	0.0070 (5)
C7	0.0308 (7)	0.0336 (8)	0.0290 (7)	−0.0030 (6)	−0.0106 (6)	0.0046 (6)
C8	0.0277 (7)	0.0332 (7)	0.0283 (7)	−0.0036 (6)	−0.0097 (6)	0.0079 (6)
C9	0.0203 (6)	0.0324 (7)	0.0244 (7)	−0.0059 (5)	−0.0050 (5)	0.0081 (5)
C10	0.0265 (7)	0.0359 (8)	0.0218 (7)	−0.0059 (6)	−0.0051 (5)	0.0087 (6)
C11	0.0283 (7)	0.0320 (7)	0.0242 (7)	−0.0041 (6)	−0.0085 (5)	0.0057 (5)
C12	0.0290 (7)	0.0361 (8)	0.0271 (7)	−0.0082 (6)	−0.0072 (6)	0.0025 (6)
C13	0.0289 (7)	0.0387 (8)	0.0284 (7)	−0.0025 (6)	−0.0073 (6)	0.0029 (6)
C14	0.0392 (9)	0.0310 (8)	0.0367 (8)	−0.0005 (6)	−0.0145 (7)	0.0017 (6)
C15	0.0390 (9)	0.0312 (8)	0.0512 (10)	−0.0094 (7)	−0.0155 (8)	0.0036 (7)
C16	0.0276 (7)	0.0358 (8)	0.0414 (9)	−0.0071 (6)	−0.0062 (6)	0.0064 (7)
C17	0.0311 (7)	0.0376 (8)	0.0216 (7)	0.0013 (6)	−0.0047 (6)	0.0058 (6)
C18	0.0490 (10)	0.0414 (9)	0.0290 (8)	−0.0012 (7)	−0.0136 (7)	0.0016 (7)
C19	0.0522 (11)	0.0545 (11)	0.0299 (8)	0.0078 (8)	−0.0175 (8)	−0.0063 (8)
C20	0.0513 (10)	0.0539 (11)	0.0212 (7)	0.0179 (8)	−0.0090 (7)	0.0028 (7)
C21	0.0428 (9)	0.0467 (10)	0.0279 (8)	0.0064 (8)	−0.0001 (7)	0.0136 (7)
C22	0.0326 (8)	0.0425 (9)	0.0275 (8)	−0.0011 (7)	−0.0026 (6)	0.0115 (6)
C23	0.0236 (10)	0.0300 (12)	0.0372 (15)	−0.0075 (8)	−0.0031 (10)	−0.0012 (9)
C24	0.0253 (9)	0.0264 (11)	0.0353 (13)	−0.0021 (8)	−0.0001 (8)	−0.0030 (9)
C25	0.0237 (14)	0.0454 (15)	0.0274 (10)	−0.0035 (11)	−0.0058 (11)	0.0037 (11)
C26	0.0272 (9)	0.0320 (10)	0.0374 (11)	−0.0071 (7)	−0.0089 (8)	−0.0006 (8)
C23'	0.009 (3)	0.045 (4)	0.028 (3)	−0.009 (3)	−0.011 (2)	0.020 (3)
C24'	0.025 (3)	0.036 (3)	0.031 (3)	−0.003 (3)	−0.004 (2)	0.001 (3)
C25'	0.023 (2)	0.033 (3)	0.030 (3)	−0.013 (2)	−0.018 (2)	0.008 (2)
C26'	0.022 (3)	0.034 (4)	0.039 (4)	−0.002 (3)	0.005 (3)	0.006 (3)
N1A	0.052 (3)	0.0387 (19)	0.052 (2)	−0.0148 (18)	−0.014 (2)	−0.0006 (18)
C2A	0.048 (2)	0.056 (3)	0.054 (3)	−0.017 (2)	−0.0080 (19)	0.001 (2)
N3A	0.056 (2)	0.064 (2)	0.053 (2)	−0.0211 (17)	−0.0173 (16)	0.0093 (17)
C4A	0.071 (4)	0.062 (5)	0.044 (3)	−0.028 (4)	−0.004 (3)	−0.007 (3)
C5A	0.052 (3)	0.045 (2)	0.060 (3)	−0.008 (2)	−0.008 (2)	−0.0175 (19)
C6A	0.062 (5)	0.071 (6)	0.055 (3)	−0.025 (4)	−0.016 (3)	0.015 (3)
N1B	0.040 (2)	0.049 (2)	0.051 (3)	−0.0091 (18)	0.000 (2)	−0.016 (2)
C2B	0.038 (3)	0.051 (3)	0.052 (3)	−0.005 (2)	0.003 (2)	0.004 (2)
N3B	0.052 (2)	0.052 (2)	0.048 (2)	−0.0133 (19)	0.0001 (18)	0.0009 (16)
C4B	0.064 (4)	0.051 (4)	0.067 (3)	0.007 (3)	−0.033 (3)	−0.025 (3)
C5B	0.052 (3)	0.084 (4)	0.071 (3)	0.016 (2)	−0.024 (2)	−0.041 (3)
C6B	0.062 (4)	0.090 (9)	0.050 (3)	−0.008 (5)	0.000 (3)	−0.011 (5)

### Geometric parameters (Å, º)

**Table:**

Fe1—N1	1.9959 (13)	C17—C18	1.399 (2)
Fe1—N1^i^	1.9960 (13)	C18—C19	1.393 (2)
Fe1—N3^i^	1.9970 (12)	C18—H18	0.9500
Fe1—N3	1.9970 (12)	C19—C20	1.385 (3)
Fe1—N2^i^	2.0008 (12)	C19—H19	0.9500
Fe1—N2	2.0008 (12)	C20—C21	1.383 (3)
N1—C4	1.381 (2)	C20—H20	0.9500
N1—C1	1.3832 (18)	C21—C22	1.397 (2)
N2—C6	1.373 (2)	C21—H21	0.9500
N2—C9	1.3814 (17)	C22—H22	0.9500
N3—C25'	1.279 (5)	C23—C24	1.380 (3)
N3—C25	1.290 (3)	C23—H23	0.9500
N3—C23	1.419 (3)	C24—H24	0.9500
N3—C23'	1.437 (5)	C25—H25	0.9500
N4—C25	1.336 (3)	C26—H26A	0.9800
N4—C24	1.360 (3)	C26—H26B	0.9800
N4—C26	1.456 (3)	C26—H26C	0.9801
N4'—C25'	1.333 (5)	C23'—C24'	1.383 (5)
N4'—C24'	1.350 (5)	C23'—H23'	0.9500
N4'—C26'	1.453 (4)	C24'—H24'	0.9500
C1—C10^i^	1.395 (2)	C25'—H25'	0.9500
C1—C2	1.443 (2)	C26'—H26D	0.9800
C2—C3	1.350 (2)	C26'—H26E	0.9800
C2—H2	0.9500	C26'—H26F	0.9800
C3—C4	1.443 (2)	N1A—C2A	1.355 (6)
C3—H3	0.9500	N1A—C5A	1.369 (6)
C4—C5	1.393 (2)	N1A—C6A	1.452 (8)
C5—C6	1.396 (2)	C2A—N3A	1.304 (6)
C5—C11	1.501 (2)	C2A—H2A	0.9500
C6—C7	1.443 (2)	N3A—C4A	1.378 (13)
C7—C8	1.353 (2)	C4A—C5A	1.358 (12)
C7—H7	0.9500	C4A—H4A	0.9500
C8—C9	1.442 (2)	C5A—H5A	0.9500
C8—H8	0.9500	C6A—H6AA	0.9800
C9—C10	1.398 (2)	C6A—H6AB	0.9800
C10—C1^i^	1.395 (2)	C6A—H6AC	0.9800
C10—C17	1.4984 (19)	N1B—C2B	1.361 (6)
C11—C16	1.390 (2)	N1B—C5B	1.362 (7)
C11—C12	1.395 (2)	N1B—C6B	1.445 (13)
C12—C13	1.391 (2)	C2B—N3B	1.292 (7)
C12—H12	0.9500	C2B—H2BB	0.9500
C13—C14	1.388 (2)	N3B—C4B	1.388 (7)
C13—H13	0.9500	C4B—C5B	1.328 (10)
C14—C15	1.388 (3)	C4B—H4B	0.9500
C14—H14	0.9500	C5B—H5B	0.9500
C15—C16	1.389 (3)	C6B—H6BA	0.9800
C15—H15	0.9500	C6B—H6BB	0.9801
C16—H16	0.9500	C6B—H6BC	0.9800
C17—C22	1.399 (2)		
			
N1—Fe1—N1^i^	180.0	C19—C18—C17	121.04 (18)
N1—Fe1—N3^i^	89.17 (5)	C19—C18—H18	119.5
N1^i^—Fe1—N3^i^	90.83 (5)	C17—C18—H18	119.5
N1—Fe1—N3	90.83 (5)	C20—C19—C18	119.92 (19)
N1^i^—Fe1—N3	89.17 (5)	C20—C19—H19	120.0
N3^i^—Fe1—N3	180.00 (3)	C18—C19—H19	120.0
N1—Fe1—N2^i^	89.66 (5)	C21—C20—C19	119.86 (16)
N1^i^—Fe1—N2^i^	90.34 (5)	C21—C20—H20	120.1
N3^i^—Fe1—N2^i^	90.09 (5)	C19—C20—H20	120.1
N3—Fe1—N2^i^	89.91 (5)	C20—C21—C22	120.51 (18)
N1—Fe1—N2	90.33 (5)	C20—C21—H21	119.7
N1^i^—Fe1—N2	89.67 (5)	C22—C21—H21	119.7
N3^i^—Fe1—N2	89.91 (5)	C21—C22—C17	120.32 (18)
N3—Fe1—N2	90.09 (5)	C21—C22—H22	119.8
N2^i^—Fe1—N2	180.00 (4)	C17—C22—H22	119.8
C4—N1—C1	105.23 (12)	C24—C23—N3	107.0 (2)
C4—N1—Fe1	127.00 (10)	C24—C23—H23	126.5
C1—N1—Fe1	127.67 (11)	N3—C23—H23	126.5
C6—N2—C9	105.26 (12)	N4—C24—C23	106.8 (2)
C6—N2—Fe1	126.95 (9)	N4—C24—H24	126.6
C9—N2—Fe1	127.73 (11)	C23—C24—H24	126.6
C25—N3—C23	105.8 (2)	N3—C25—N4	112.9 (3)
C25'—N3—C23'	99.9 (5)	N3—C25—H25	123.6
C25'—N3—Fe1	140.9 (4)	N4—C25—H25	123.6
C25—N3—Fe1	129.68 (18)	N4—C26—H26A	109.5
C23—N3—Fe1	124.41 (14)	N4—C26—H26B	109.5
C23'—N3—Fe1	119.3 (4)	H26A—C26—H26B	109.5
C25—N4—C24	107.6 (2)	N4—C26—H26C	109.5
C25—N4—C26	125.9 (2)	H26A—C26—H26C	109.5
C24—N4—C26	126.5 (2)	H26B—C26—H26C	109.5
C25'—N4'—C24'	113.0 (6)	C24'—C23'—N3	114.8 (7)
C25'—N4'—C26'	119.9 (7)	C24'—C23'—H23'	122.6
C24'—N4'—C26'	127.0 (7)	N3—C23'—H23'	122.6
N1—C1—C10^i^	125.77 (14)	N4'—C24'—C23'	98.4 (7)
N1—C1—C2	110.08 (14)	N4'—C24'—H24'	130.8
C10^i^—C1—C2	124.14 (14)	C23'—C24'—H24'	130.8
C3—C2—C1	107.41 (14)	N3—C25'—N4'	113.7 (6)
C3—C2—H2	126.3	N3—C25'—H25'	123.2
C1—C2—H2	126.3	N4'—C25'—H25'	123.2
C2—C3—C4	106.69 (15)	N4'—C26'—H26D	109.5
C2—C3—H3	126.7	N4'—C26'—H26E	109.5
C4—C3—H3	126.7	H26D—C26'—H26E	109.5
N1—C4—C5	125.81 (14)	N4'—C26'—H26F	109.5
N1—C4—C3	110.58 (13)	H26D—C26'—H26F	109.5
C5—C4—C3	123.61 (15)	H26E—C26'—H26F	109.5
C4—C5—C6	123.75 (15)	C2A—N1A—C5A	106.7 (4)
C4—C5—C11	117.86 (13)	C2A—N1A—C6A	125.9 (6)
C6—C5—C11	118.37 (13)	C5A—N1A—C6A	127.4 (5)
N2—C6—C5	126.04 (13)	N3A—C2A—N1A	112.0 (4)
N2—C6—C7	110.72 (13)	N3A—C2A—H2A	124.0
C5—C6—C7	123.18 (15)	N1A—C2A—H2A	124.0
C8—C7—C6	106.68 (14)	C2A—N3A—C4A	105.3 (6)
C8—C7—H7	126.7	C5A—C4A—N3A	109.9 (9)
C6—C7—H7	126.7	C5A—C4A—H4A	125.1
C7—C8—C9	107.00 (13)	N3A—C4A—H4A	125.1
C7—C8—H8	126.5	C4A—C5A—N1A	106.1 (7)
C9—C8—H8	126.5	C4A—C5A—H5A	127.0
N2—C9—C10	125.56 (14)	N1A—C5A—H5A	127.0
N2—C9—C8	110.32 (13)	N1A—C6A—H6AA	109.5
C10—C9—C8	124.12 (13)	N1A—C6A—H6AB	109.5
C1^i^—C10—C9	123.47 (13)	H6AA—C6A—H6AB	109.5
C1^i^—C10—C17	118.17 (14)	N1A—C6A—H6AC	109.5
C9—C10—C17	118.33 (14)	H6AA—C6A—H6AC	109.5
C16—C11—C12	118.56 (15)	H6AB—C6A—H6AC	109.5
C16—C11—C5	120.91 (14)	C2B—N1B—C5B	105.1 (5)
C12—C11—C5	120.53 (14)	C2B—N1B—C6B	126.0 (7)
C13—C12—C11	120.91 (15)	C5B—N1B—C6B	128.9 (7)
C13—C12—H12	119.5	N3B—C2B—N1B	113.0 (6)
C11—C12—H12	119.5	N3B—C2B—H2BB	123.5
C14—C13—C12	119.83 (15)	N1B—C2B—H2BB	123.5
C14—C13—H13	120.1	C2B—N3B—C4B	104.3 (6)
C12—C13—H13	120.1	C5B—C4B—N3B	109.9 (6)
C15—C14—C13	119.73 (16)	C5B—C4B—H4B	125.0
C15—C14—H14	120.1	N3B—C4B—H4B	125.0
C13—C14—H14	120.1	C4B—C5B—N1B	107.6 (5)
C14—C15—C16	120.17 (16)	C4B—C5B—H5B	126.2
C14—C15—H15	119.9	N1B—C5B—H5B	126.2
C16—C15—H15	119.9	N1B—C6B—H6BA	109.5
C15—C16—C11	120.78 (15)	N1B—C6B—H6BB	109.5
C15—C16—H16	119.6	H6BA—C6B—H6BB	109.5
C11—C16—H16	119.6	N1B—C6B—H6BC	109.5
C22—C17—C18	118.34 (14)	H6BA—C6B—H6BC	109.5
C22—C17—C10	121.53 (15)	H6BB—C6B—H6BC	109.5
C18—C17—C10	120.11 (15)		
			
C4—N1—C1—C10^i^	179.49 (15)	C14—C15—C16—C11	−0.5 (3)
Fe1—N1—C1—C10^i^	−4.0 (2)	C12—C11—C16—C15	−0.9 (2)
C4—N1—C1—C2	−1.29 (18)	C5—C11—C16—C15	178.97 (14)
Fe1—N1—C1—C2	175.20 (11)	C1^i^—C10—C17—C22	123.17 (17)
N1—C1—C2—C3	0.9 (2)	C9—C10—C17—C22	−58.6 (2)
C10^i^—C1—C2—C3	−179.91 (16)	C1^i^—C10—C17—C18	−58.1 (2)
C1—C2—C3—C4	−0.1 (2)	C9—C10—C17—C18	120.08 (17)
C1—N1—C4—C5	−179.10 (15)	C22—C17—C18—C19	0.0 (3)
Fe1—N1—C4—C5	4.4 (2)	C10—C17—C18—C19	−178.75 (16)
C1—N1—C4—C3	1.26 (17)	C17—C18—C19—C20	0.9 (3)
Fe1—N1—C4—C3	−175.27 (11)	C18—C19—C20—C21	−0.8 (3)
C2—C3—C4—N1	−0.8 (2)	C19—C20—C21—C22	−0.2 (3)
C2—C3—C4—C5	179.59 (16)	C20—C21—C22—C17	1.1 (3)
N1—C4—C5—C6	−2.7 (2)	C18—C17—C22—C21	−1.0 (2)
C3—C4—C5—C6	176.88 (15)	C10—C17—C22—C21	177.77 (15)
N1—C4—C5—C11	178.57 (14)	C25—N3—C23—C24	0.9 (3)
C3—C4—C5—C11	−1.8 (2)	Fe1—N3—C23—C24	177.80 (16)
C9—N2—C6—C5	−177.47 (14)	C25—N4—C24—C23	−0.6 (3)
Fe1—N2—C6—C5	0.1 (2)	C26—N4—C24—C23	176.1 (2)
C9—N2—C6—C7	−0.16 (16)	N3—C23—C24—N4	−0.2 (3)
Fe1—N2—C6—C7	177.38 (10)	C23—N3—C25—N4	−1.3 (4)
C4—C5—C6—N2	0.4 (2)	Fe1—N3—C25—N4	−178.00 (15)
C11—C5—C6—N2	179.09 (13)	C24—N4—C25—N3	1.2 (4)
C4—C5—C6—C7	−176.61 (14)	C26—N4—C25—N3	−175.5 (2)
C11—C5—C6—C7	2.1 (2)	C25'—N3—C23'—C24'	3.6 (13)
N2—C6—C7—C8	−0.60 (17)	Fe1—N3—C23'—C24'	−177.6 (7)
C5—C6—C7—C8	176.80 (14)	C25'—N4'—C24'—C23'	2.9 (12)
C6—C7—C8—C9	1.07 (17)	C26'—N4'—C24'—C23'	−177.8 (9)
C6—N2—C9—C10	−179.52 (14)	N3—C23'—C24'—N4'	−4.0 (12)
Fe1—N2—C9—C10	3.0 (2)	C23'—N3—C25'—N4'	−1.5 (12)
C6—N2—C9—C8	0.84 (15)	Fe1—N3—C25'—N4'	−179.9 (4)
Fe1—N2—C9—C8	−176.68 (9)	C24'—N4'—C25'—N3	−0.9 (14)
C7—C8—C9—N2	−1.24 (17)	C26'—N4'—C25'—N3	179.7 (8)
C7—C8—C9—C10	179.11 (14)	C5A—N1A—C2A—N3A	1.0 (5)
N2—C9—C10—C1^i^	−0.1 (2)	C6A—N1A—C2A—N3A	179.4 (5)
C8—C9—C10—C1^i^	179.49 (14)	N1A—C2A—N3A—C4A	−0.7 (7)
N2—C9—C10—C17	−178.22 (13)	C2A—N3A—C4A—C5A	0.2 (9)
C8—C9—C10—C17	1.4 (2)	N3A—C4A—C5A—N1A	0.4 (9)
C4—C5—C11—C16	−97.78 (18)	C2A—N1A—C5A—C4A	−0.9 (7)
C6—C5—C11—C16	83.43 (18)	C6A—N1A—C5A—C4A	−179.2 (7)
C4—C5—C11—C12	82.09 (18)	C5B—N1B—C2B—N3B	−1.6 (6)
C6—C5—C11—C12	−96.70 (17)	C6B—N1B—C2B—N3B	176.7 (8)
C16—C11—C12—C13	1.4 (2)	N1B—C2B—N3B—C4B	1.9 (7)
C5—C11—C12—C13	−178.47 (13)	C2B—N3B—C4B—C5B	−1.6 (9)
C11—C12—C13—C14	−0.5 (2)	N3B—C4B—C5B—N1B	0.7 (9)
C12—C13—C14—C15	−0.9 (2)	C2B—N1B—C5B—C4B	0.4 (7)
C13—C14—C15—C16	1.4 (2)	C6B—N1B—C5B—C4B	−177.7 (9)

Symmetry code: (i) −*x*+1, −*y*+1, −*z*+1.

### Hydrogen-bond geometry (Å, º)

**Table:**

*D*—H···*A*	*D*—H	H···*A*	*D*···*A*	*D*—H···*A*
C19—H19···N3*A*^ii^	0.95	2.55	3.478 (4)	164

Symmetry code: (ii) *x*+1, *y*, *z*+1.
