# Supplementary material for: Crystal structure of bis­(1-methyl-1H-imidazole-κN 3)(5,10,15,20-tetra­phenyl­porphyrinato-κ4 N)iron(II)–1-methyl-1H-imidazole (1/2)
Source: Acta Crystallogr E Crystallogr Commun. 2015 Feb 11;71(Pt 3):m57–8. doi: 10.1107/S2056989015002364 (PMC4350711; doi:10.1107/S2056989015002364)
Supplement: Supplementary file 4 [file e-71-00m57-Isup4.docx]

Comment

Bis-histidine coordinated hemes are present in a number of cytochrome *b* complexes, and are known to be involved in electron transfer processes (Xia *et al.*, 1997). The parallel and perpendicular relative orientations of the histidine ligands are believed to have correlations with the spectroscopic properties of the proteins (Walker, 2004). As models of bis-histidine coordinated cytochrome *b*, several cationic bis-imidazole coordinated porphyrin complexes have been synthesized and their structures have been determined (Safo *et al.*, 1991). Here, we report the molecular structure of a neutral bis-imidazole coordinated iron(II) complex, Fe(TPP)(1-MeIm)_2_^.^2(1-MeIm). The molecular structure of the titled compound is shown in figure.1. The porphyrin complex was located on an inversion center. The 1-methylimidazole groups bonded to the metal and the unbound 1-methylimidazoles were disordered. The occupancies of the metal-bound ligands were refined to 0.789
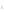
(4) and 0.211
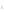
(4) for the unprimed and primed atoms. The occupancies of the unbound 1-methylimidazoles were refined to 0.519
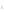
(4) and 0.481
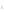
(4) for the A and B molecules. The average Fe-Np bond distance is 1.9959
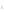
(13)
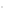
Å and the axial Fe-N_Im_ distance is 1.9989
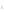
(12)
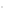
Å, suggesting a low-spin ferrous center (Scheidt & Reid, 1981). The two 1-MeIm planes are mutually parallel. The projection of the axial ligand has a dihedral angle of 28.04 ° with the closest Fe-Np bond.

Synthesize and crystallization

The Fe(TPP)(1-MeIm)_2_^.^2(1-MeIm) was obtained serendipitously as follows: To a 10
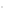
ml CH_2_Cl_2_ solution of (TPP)FeCl (0.010
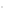
g, 0.014
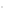
mmol) was added N-hydroxyamphetamine (7
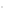
mg, 46.3
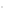
mmol) and 1-MeIm (0.05
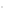
ml, 29.0
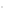
mmol) under nitrogen. The color of the solution changed from brown to reddish purple during a 6
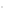
h period. The solution was dried under reduced pressure. The residue was dissolved in CH_2_Cl_2_ and filtered, and an equal volume of hexane was added. A red plate-shaped crystal grew from the slow evaporation of this mixture at room temperature under nitrogen.
