# Supplementary figures and images for: Crystal structure of bis­(1-methyl-1H-imidazole-κN 3)(5,10,15,20-tetra­phenyl­porphyrinato-κ4 N)iron(II)–1-methyl-1H-imidazole (1/2)
Source: Acta Crystallogr E Crystallogr Commun. 2015 Feb 11;71(Pt 3):m57–8. doi: 10.1107/S2056989015002364 (PMC4350711; doi:10.1107/S2056989015002364)

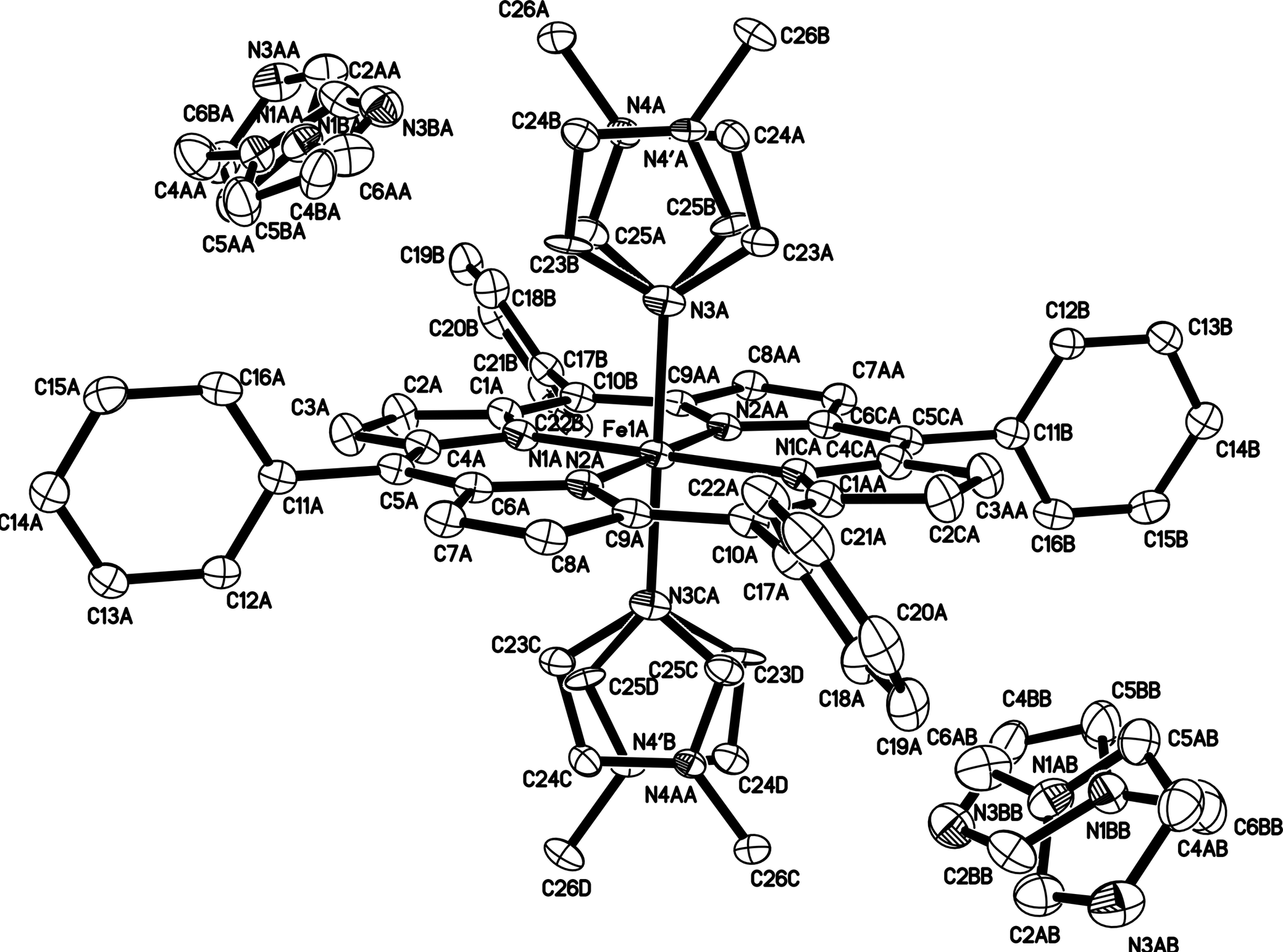

Supplement: Supplementary file 5 [file e-71-00m57-fig1.tif]
